# Comparison of laparoscopic sacrocolpopexy with vaginal reconstructive procedures and abdominal sacrocolpopexy for the surgical management of vaginal vault prolapse: a systematic review and meta-analysis

**DOI:** 10.3389/fmed.2023.1269214

**Published:** 2023-09-12

**Authors:** Răzvan Ciortea, Maria-Patricia Roman, Andrei Mihai Măluțan, Carmen Elena Bucuri, Cristina Mihaela Ormindean, Ionel Daniel Nati, Dan Mihu

**Affiliations:** ^1^Mother and Child Department, “Iuliu Hațieganu” University of Medicine and Pharmacy, Cluj-Napoca, Romania; ^2^2nd Obstetrics and Gynaecology Clinical Section, Cluj County Emergency Clinical Hospital, Cluj-Napoca, Romania; ^3^Military Emergency Hospital “Dr. Constantin Papilian”, Cluj-Napoca, Romania

**Keywords:** apical prolapse, vault prolapse, sacrocolpopexy, sacrospinous fixation, vaginal mesh, randomized trial, cohort study, meta-analysis

## Abstract

**Introduction:**

Vaginal vault prolapse, also known as apical prolapse, is a distressing condition that may affect women following hysterectomy, necessitating surgical intervention when conservative measures prove ineffective. The surgical management of apical compartment prolapse includes procedures such as laparoscopic sacrocolpopexy (LSCP), abdominal sacrocolpopexy (ASCP) or vaginal reconstructive procedures (VRP). This systematic review and meta-analysis aims to compare the outcomes of these interventions.

**Methods:**

A comprehensive search of electronic databases was conducted to identify eligible studies. Fourteen studies comprising a total of 1,289 women were included. The selected studies were analyzed to evaluate outcomes such as duration of surgery, length of hospital stay, blood loss, complication rates, and patient satisfaction.

**Results:**

LSCP did not demonstrate significant advantages over VRP in terms of perioperative or long-term outcomes. However, when compared to ASCP, LSCP showed shorter hospital stay, reduced blood loss, decreased postoperative pain, and lower rates of ileus.

**Discussion:**

This systematic review contributes to evidence-based decision-making for the surgical treatment of vaginal vault prolapse. While LSCP did not exhibit substantial benefits over VRP, it emerged as a preferable option compared to ASCP due to shorter hospital stays and reduced postoperative complications. The findings from this study provide valuable insights for clinicians and patients in selecting the most appropriate surgical approach for vaginal vault prolapse. However, future research should focus on long-term follow-ups, standardizing outcomes, and outcome measures, and evaluating cost-effectiveness to further enhance clinical practice.

## 1. Introduction

Hysterectomy remains a common gynaecological procedure, although its prevalence has been decreasing in some countries in recent years due to advancements in conservative treatment options, increased utilization of minimally invasive techniques, and a shift towards more organ-preserving approaches (1).

Vaginal vault prolapse, a condition characterized by the descent of the vaginal apex following hysterectomy, has an overall prevalence ranging from 0.2 to 43% (2–5) and represents a significant concern for many women worldwide. This distressing condition can lead to a multitude of symptoms, including pelvic pressure, discomfort during sexual intercourse, low back pain, voiding dysfunction, and an overall diminished quality of life (6). When conservative measures such as pelvic floor physiotherapy, pessary use, or lifestyle changes fail, surgical intervention often becomes a necessity to restore pelvic support, alleviate symptoms, and enhance a patient’s well-being. Currently, it is estimated that 23% of women with symptomatic apical prolapse eventually undergo surgical intervention (7, 8).

Surgical management options for apical prolapse include various procedures, such as sacrocolpopexy (laparoscopic, robotic, or abdominal), sacrospinous ligament fixation, uterosacral ligament suspension, iliococcygeus fixation, as well as transvaginal mesh procedures. Advancements in minimally invasive surgical techniques, such as laparoscopic approach, have become more and more accessible and expanded the options for surgical treatment, allowing for potentially faster recovery times and reduced postoperative morbidity. Laparoscopic sacrocolpopexy (LSCP) has emerged as the current gold standard for the surgical treatment of apical pelvic organ prolapse (9–12).

The specific surgical technique choice depends on factors such as the severity of prolapse, the patient’s overall health, surgeon expertise, and individualized treatment goals. As the medical community strives to optimize patient outcomes, it becomes crucial to thoroughly explore and compare these surgical techniques to determine their respective benefits, limitations, and overall efficacy.

This article aims to provide a comprehensive, pooled analysis and comparison of three commonly used surgical techniques for the treatment of vaginal vault prolapse, namely LSCP, abdominal sacrocolpopexy (ASCP), and vaginal reconstructive procedures (VRP). By assessing their outcomes, complications, and patient satisfaction rates, we seek to offer clinicians and patients alike a detailed understanding of the advantages and potential drawbacks of each technique.

By comparing LSCP with both VRP and ASCP, this study aimed to evaluate the differences in surgical outcomes, including improvement in symptoms, complication rates, length of hospital stay, operative time, and patient satisfaction. These outcomes were selected to provide a comprehensive assessment of the comparative effectiveness and safety of different surgical approaches. The inclusion of multiple comparators allows the exploration of the advantages and disadvantages of each technique, providing valuable insights in making informed decisions regarding the most appropriate surgical management for vaginal vault prolapse.

## 2. Methods

### 2.1. Study design

This systematic review and meta-analysis included randomised controlled trials (RCT) and retrospective or prospective cohort studies reporting outcomes of surgical interventions performed for apical prolapse after hysterectomy. Systematic reviews, case reports, letters to editor, commentaries, educational articles, study protocols, non-comparative studies were excluded from our analysis. The identified studies were selected and reported in accordance with the updated Preferred Reporting Items for Systematic Reviews and Meta-Analyses (PRISMA) guidelines (13).

### 2.2. Participants

Comparative studies including women of any age or ethnicity suffering from apical prolapse who opted for surgical management of their condition were included.

### 2.3. Comparators

In this systematic review and meta-analysis, we aimed to compare the outcomes of LSCP with two alternative surgical procedures for the treatment of vaginal vault prolapse, namely VRP and ASCP. The selection of these comparators was based on their frequent utilization in clinical practice, the presence of pertinent studies for inclusion in our analysis, and the recent trend favouring minimally invasive surgical approaches.

It is worth emphasizing that the choice of comparators in this study was also guided by the current evidence, that highlighted a lack of comparisons of LSCP, VRP, and ASCP in women with a history of hysterectomy, suffering from prolapse of the apical compartment. Hence, through a meticulous and systematic search process, our objective was to identify studies that directly compared LSCP with either VRP or ASCP technique in this specific patient population. This approach aimed to ensure that the findings of our study would be relevant, applicable in clinical practice, and fill an important gap in the current clinical literature.

### 2.4. Systematic review protocol

This study has been registered in the PROSPERO International Prospective Register of Systematic Reviews (registration number CRD42023441528).

### 2.5. Search strategy and data sources

A comprehensive literature search was conducted using electronic databases (Medline, Embase, Cochrane Library, Scopus, Google Scholar) from inception to July 2023 to identify relevant studies. The search strategy included a combination of the following keywords and Medical Subject Headings (MeSH) terms: “apical prolapse,” “vault prolapse,” “middle compartment prolapse,” “laparoscopic sacrocolpopexy,” “randomised controlled trial,” “RCT,” “randomized trial.” The terms were combined using logical operators such as ‘AND’ and ‘OR’ to retrieve relevant results. The records were deduplicated. Additional sources, such as reference lists of relevant articles were also searched to ensure comprehensive coverage of the literature through “snowballing technique.” This technique allowed the expansion of the initial list of selected articles by following the chain of citations and references to uncover more potentially relevant studies. The search process was not restricted based on language, allowing for the inclusion of studies published in any language.

### 2.6. Data extraction

A standardized data extraction form was used to extract relevant information from the included studies. The following data were collected: study characteristics (first author, publication year, study design, sample size, hysterectomy status) and type of surgical procedures that have been compared. Data extraction was performed independently by two reviewers, and any discrepancies were resolved through discussion and consensus within the research team. Among the three surgical techniques compared, LSCP was considered the primary comparator. The other two techniques were VRP and ASCP. Studies that assessed the same surgical procedures were grouped together for the purpose of pooled analyses.

### 2.7. Data analysis

The statistical analyses for dichotomous and continuous data were conducted using Review Manager 5.4. The effect size of different surgical interventions for apical prolapse was presented as an odds ratio with a corresponding 95% confidence interval (CI) for dichotomous variables or as mean difference with a 95% CI for continuous data. The degree of heterogeneity among studies was assessed using the I^2^ statistic. When substantial homogeneity was observed (*I*^2^ < 50%), pooled summary statistics were calculated using fixed-effects models. In instances of notable heterogeneity (I^2^ > 50%), random-effects models were utilized.

## 3. Results

### 3.1. Prisma diagram

**Figure 1:**
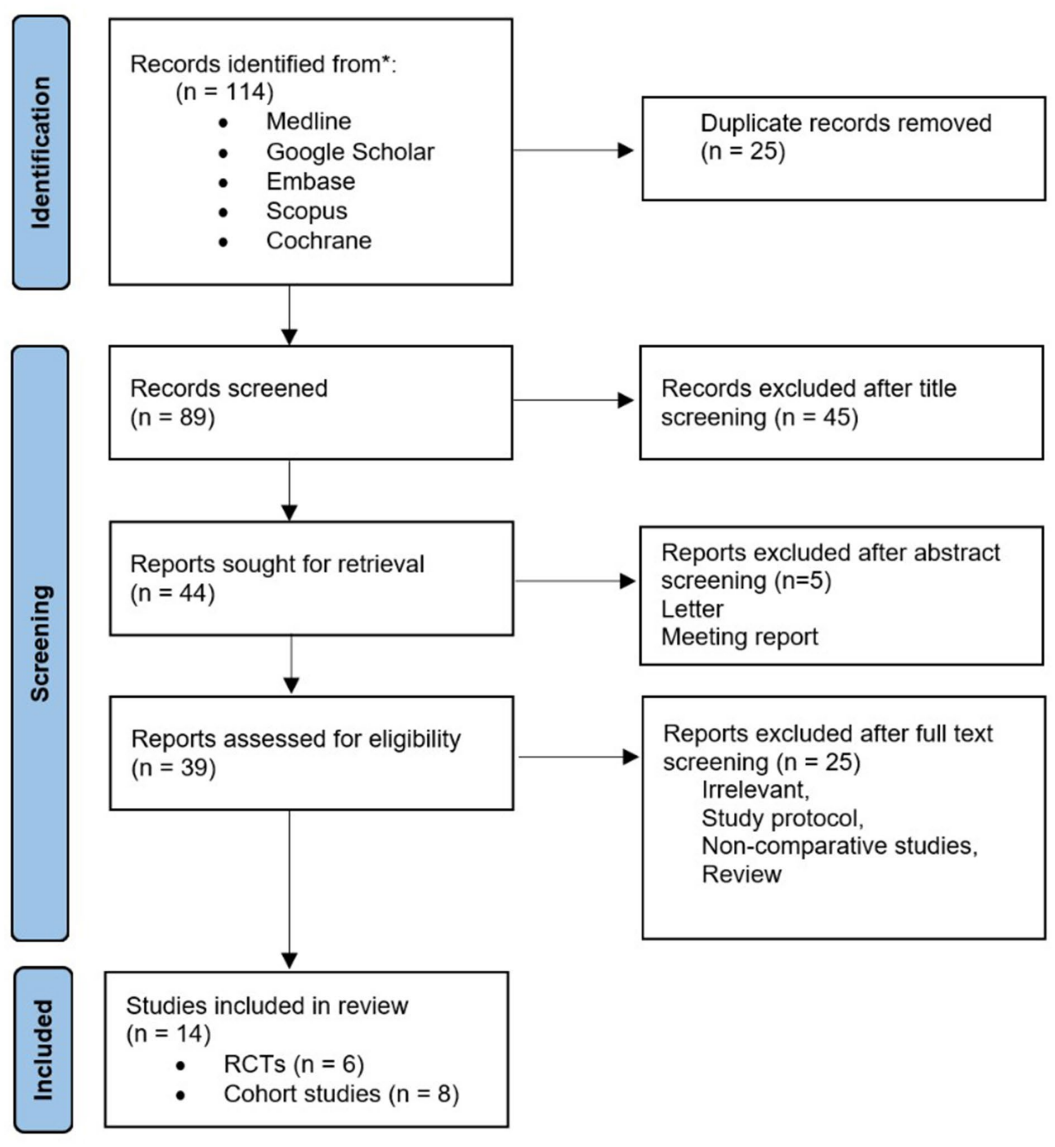
Prisma flow diagram.

Following comprehensive searches of multiple databases, a total of 114 studies reporting outcomes of three distinct surgical techniques for vaginal vault prolapse were initially identified and considered potentially eligible for inclusion in this systematic review and meta-analysis. After deduplication of records, 89 studies remained and were screened by title. Of those, 45 were excluded. The remaining 44 articles were sought for retrieval and screened by abstracts. Finally, after assessing eligibility based on the full-text articles, a total of 14 studies including 1,289 women were deemed eligible for the analysis. In one study, two arms were included, consisting of one RCT and one prospective cohort study (8). The inclusion process and the number of studies ultimately meeting the eligibility criteria are summarized in the Prisma Flow Diagram (Figure 1).

### 3.2. Study selection and characteristics

The characteristics of the included studies are presented in Table 1.

**Table 1:** Characteristics of the included studies.

Study	Publication year	Sample size	Study design	Hysterectomy status	Laparoscopic sacrocolpopexy	Abdominal sacrocolpopexy	Vaginal reconstructive procedure
Marcickiewicz et al. (14)	2007	111	Retrospective cohort	History of hysterectomy	x		x
van Oudheusden et al. (8)^*^	2023	64	RCT	History of hysterectomy	x		x
van Oudheusden et al. (8)^**^		115	Prospective cohort		x		x
Maher et al. (15)	2012	108	RCT	History of hysterectomy	x		x
Withagen et al. (16)	2013	97	Prospective cohort	History of hysterectomy	x		x
Okcu et al. (17)	2021	65	Prospective cohort	Concurrent hysterectomy during apical prolapse surgery	x	x	x
Costantini et al. (18)	2016	120	RCT	Concurrent hysterectomy during apical prolapse surgery	x	x	
Freeman et al. (19)	2013	53	RCT	History of hysterectomy	x	x	
van Oudheusden et al. (20)	2022	41	RCT	History of hysterectomy	x	x	
Coolen et al. (21)	2017	74	RCT	History of hysterectomy	x	x	
Coolen et al. (22)	2013	85	Prospective cohort	History of hysterectomy	x	x	
Klauschie et al. (23)	2009	84	Retrospective cohort	History of hysterectomy or concurrent hysterectomy during apical prolapse surgery	x	x	
Paraiso et al. (24)	2005	117	Retrospective cohort	History of hysterectomy	x	x	
Poovathai et al. (25)	2023	50	Prospective cohort	History of hysterectomy	x	x	
Cho et al. (26)	2022	105	Retrospective cohort	Concurrent hysterectomy during apical prolapse surgery	x	x	

With the exception of one study (27), all the studies included in this review directly compared the outcomes of only two surgical techniques for vaginal vault prolapse. Okcu et al. conducted a study that compared the outcomes of three different procedures, namely LSCP, ASCP, and VRP (17). Among the included studies, five studies conducted comparisons between LSCP and VRP, while ten studies focused on comparing LSCP with ASCP.

Meta-analyses were conducted for those outcomes that were consistently reported across at least three primary studies in a comparable fashion.

### 3.3. Synthesized findings

**Figure 2:**
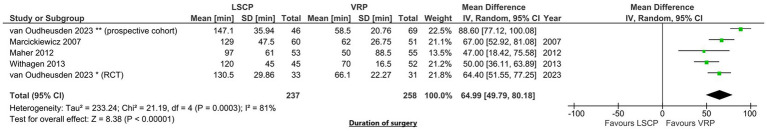
Forest plot of duration of surgery, the sole outcome that showed statistical significance when comparing LSCP and VRP.

For studies comparing LSCP with VRP, meta-analyses were carried out for the following outcomes: duration of surgery, length of hospital stay, blood loss, pelvic organ prolapse at follow-up, urinary symptoms at follow-up, dyspareunia, and Urogenital Distress Inventory (UDI) scores. Random effects forest plots (Figure 2) showed that only the duration of surgery significantly differ among those groups, LSCP lasting significantly longer than VRP (*p* < 0.0001).

**Figure 3:**
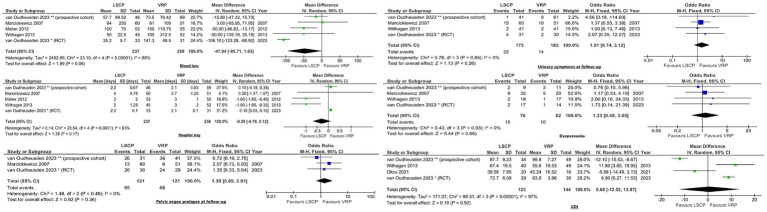
Forest plots displaying outcomes comparing LSCP with VRP, where statistical significance was not observed.

The forest plots for the outcomes that did not yield significant differences between LSCP and VRP are shown in Figure 3.

**Figure 4:**
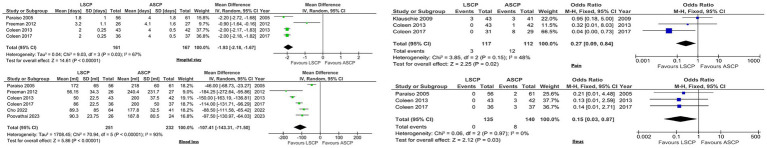
Forest plots illustrating outcomes that exhibited significant differences among LSCP and ASCP group.

Meta-analyses were conducted to assess the following outcomes when comparing LSCP with ASCP: duration of surgery, length of hospital stay, blood loss, haemorrhage, bladder/bowel injury, urinary symptoms at follow-up, pain, wound infection, ileus rates, pulmonary embolism (PE) /deep vein thrombosis (DVT), UDI and Incontinence Impact Questionnaire (IIQ) scores. The meta-analyses showed significant differences in terms of hospital stay (*p* < 0.00001), blood loss (*p* < 0.00001), pain (*p* = 0.02) and rates of postoperative ileus (*p* = 0.03). Women in the LSCP group had shorter hospital stay, less blood loss and pain, as well as lower rates of ileus (Figure 4).

**Figure 5:**
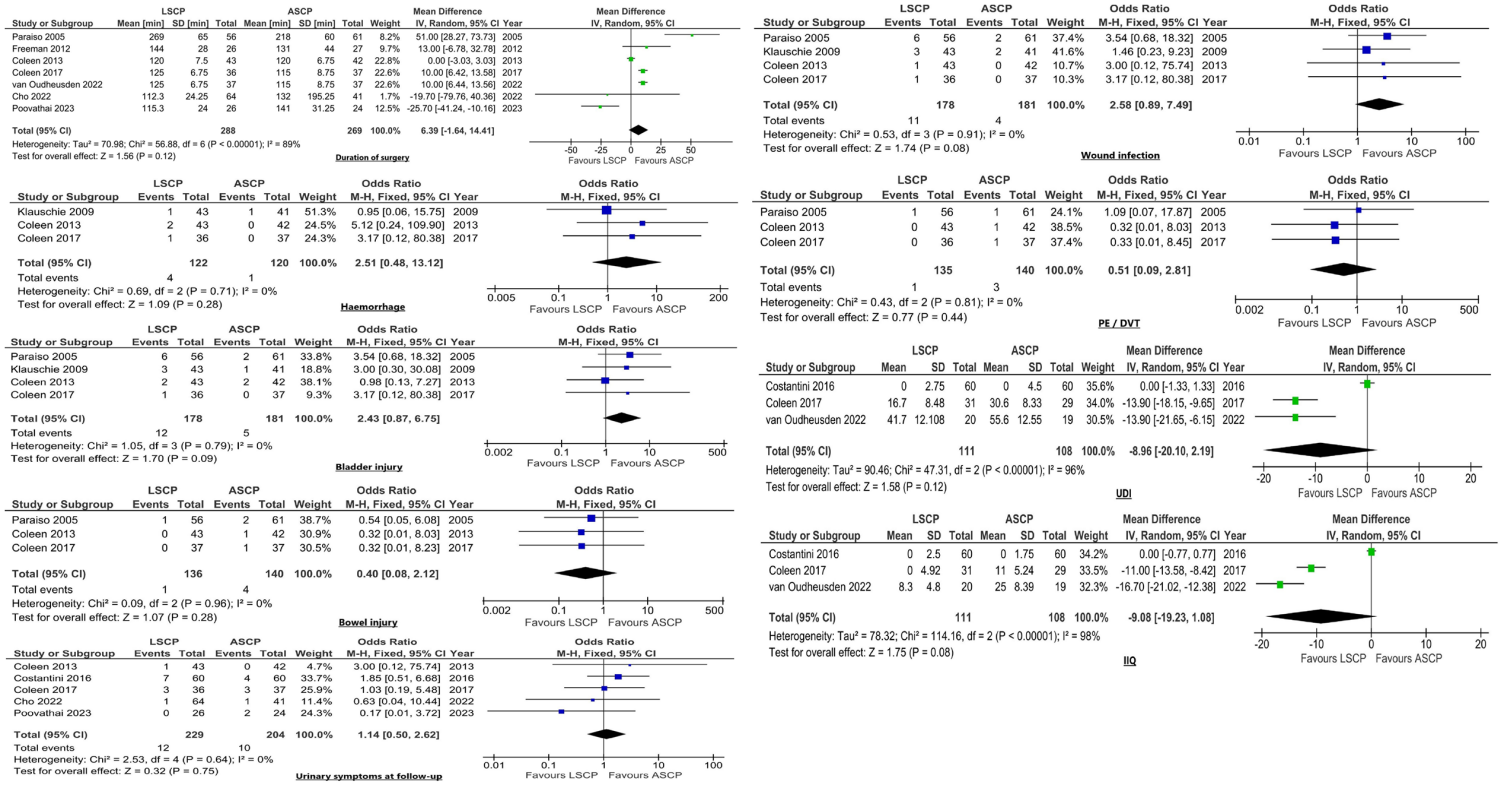
Forest plots depicting the outcomes that showed no significant differences among the groups (LSCP vs. ASCP).

For the remaining outcomes mentioned above, no statistically significant differences were found among the groups (Figure 5).

### 3.4. Assessment of risk of bias

This meta-analysis encompasses evidence derived from both RCT, which are designed to minimize systematic errors, and from non-randomized studies, which may be more susceptible to bias. To evaluate the risk of bias as well as the quality of the included studies we used Critical Appraisals Skills Programme (CASP) tools for randomised and cohort studies (28, 29). Two researchers critically appraised the included studies. Any disagreements were resolved through discussion within the research team. Tables 2, 3 present CASP criteria for RCT and cohort studies.

**Table 2:** CASP criteria for RCT.

CASP criteria/RCT	Coolen et al. (21)	Costantini et al. (18)	Freeman et al. (19)	Maher et al. (15)	van Oudheusden et al. (8)	van Oudheusden et al. (20)
Did the trial address a clearly focused issue?	y	y	y	y	y	y
Was the assignment of patients to treatments randomised?	y	y	y	y	y	y
Were all of the patients who entered the trial properly accounted for at its conclusion?	n	n	n	y	n	n
Were patients, health workers and study personnel ‘blind’ to treatment?	n	n	n	n	n	n
Were the groups similar at the start of the trial?	y	y	ct	y	y	y
Apart from the experimental intervention, did each study group receive the same level of care (that is, were they treated equally)?	y	y	y	y	y	y
Were the effects of intervention reported comprehensively?	y	y	y	y	y	y
Was the precision of the estimate of the intervention or treatment effect reported?	y	n	y	y	n	y
Do the benefits of the experimental intervention outweigh the harms and costs?	y	y	y	y	y	y
Can the results be applied to your local population/in your context?	y	y	y	y	y	y
Would the experimental intervention provide greater value to the people in your care than any of the existing interventions?	y	y	y	y	y	y

Y, yes; n, no; ct, cannot tell.

**Table 3:** CASP criteria for cohort studies.

CASP criteria/cohort study	Marcickiewicz et al. (14)	Klauschie et al. (23)	Coolen et al. (22)	Withagen et al. (16)	Okcu et al. (17)	Paraiso et al. (24)	Poovathai et al. (25)	Cho et al. (26)
Did the study address a clearly focused issue?	y	y	y	y	y	y	y	y
Was the cohort recruited in an acceptable way?	y	y	y	y	y	y	n	y
Was the exposure accurately measured to minimise bias?	n	n	y	y	y	y	n	n
Was the outcome accurately measured to minimise bias?	n	y	y	y	y	y	y	y
Have the authors identified all important confounding factors?	y	y	y	y	y	y	n	y
Have they taken account of the confounding factors in the design and/or analysis?	y	y	y	y	y	y	n	y
Was the follow up of subjects complete enough?	n	y	n	y	ct	n	n	n
Was the follow up of subjects long enough?	y	y	n	y	y	n	n	y
Do you believe the results?	y	y	y	y	y	y	y	y
Can the results be applied to the local population?	y	y	y	y	y	y	y	y
Do the results of this study fit with other available evidence?	y	y	y	y	y	y	y	y

As indicated in Table 2, blinding of both patients and medical professionals was unfeasible in the included RCTs due to the specific types of incisions required for each type of surgical procedure. Due to this valid rationale, none of the trials met the “blinding” criteria. Additionally, the presence of lost-to-follow-up patients contributed to another criterion that could not be met in terms of study quality by five out of six included RCTs. Another identified source of bias pertained to the precision of the reported estimate of the intervention or treatment effect.

Table 3 reveals that in the cohort studies, there were certain concerns raised regarding the accuracy of exposure measurement and the adequacy of follow-up length and completeness. Isolated concerns regarding potential confounding factors and the manner of cohort recruitment were also identified and highlighted in Table 3.

## 4. Discussion

### 4.1. Summary of main findings

A variety of surgical approaches have been developed, optimised, and implemented to surgically treat vaginal apical prolapse (30). These include LSCP, open ASCP, as well as VRP. Each of these approaches offers distinct benefits compared to one another. Given that the LSCP has achieved the status of the current gold standard, primarily due to its high cure rates (31, 32), it is reasonable to synthesize data on this procedure as well as comparisons between LSCP and alternative surgical techniques.

This systematic review and meta-analysis offer valuable insights into the differences of pooled outcomes of LSCP compared to ASCP or VRP. Our analyses showed that LSCP does not have significant perioperative or long-term advantages over VRP performed for vaginal vault prolapse. Moreover, when compared with LSCP, VRP were associated with a significantly shorter duration of surgery (*p* < 0.0001). These data could render VRP particularly advantageous for elderly women with underlying health conditions. Published data supports the benefits of shorter operative time that include reduced anaesthesia time and surgical risks, enhanced patient comfort, faster recovery, reduced resource requirements, and improved surgical throughput (33). Since the operating time was longer for LSCP, one would expect more blood loss in those cases. However, our study showed that was not the case, as blood loss showed lower values in the LSCP group, although this difference did not reach statistical significance. It is plausible that reduced blood loss in case of laparoscopic procedures can be attributed to better visualisation, easier tissue manipulation and access (34, 35). Furthermore, the choice of anaesthesia may potentially influence blood loss outcomes. Nonetheless, it is essential to highlight that our review did not include data pertaining to the specific anaesthesia types employed for each procedure as not all the primary studies included documented this information.

Outcomes such as hospital stay, pelvic organ prolapse or urinary symptoms at follow-up, dyspareunia and UDI scores did not significantly differ between LSCP and VRP groups. However, some of these outcomes such as hospital stay showed significant differences between groups in individual studies (15, 17). The discrepancy between individual study results and pooled analyses can be attributed to various factors, such as study sample sizes, variability in patient populations, and study design differences.

On the other hand, this meta-analysis showed that when compared to ASCP, patients undergoing LSCP had significantly shorter hospital stay, less blood loss and pain, as well as lower ileus rates (*p* < 0.05). Most of the primary studies included in this analysis reported similar results, with the exception of one study (23). Klauschie et al. reported similar levels of pain in both ASCP and LSCP groups (23). However, when pooling multiple studies in a meta-analysis, the increased sample size enhances the statistical power to detect significant differences between the two surgical techniques.

Furthermore, this study indicated that the LSCP groups exhibited lower IIQ total scores, wound infection and bladder injury rates compared to the ASCP groups, but statistical significance was not achieved for those outcomes. Although individual studies might have shown significant differences between those outcomes, pooled analyses allowed the combination of data from multiple studies, mitigating the impact of individual study variations and providing a more comprehensive assessment of the overall effect.

Comparing these findings with those of other meta-analyses is challenging due to the variations in the inclusion criteria. Most reviews or meta-analyses evaluating surgical approaches for apical pelvic organ prolapse have included a broader population, encompassing both women post hysterectomy and those with a uterus (30, 36, 37) while our study included only women without a uterus. As a result, direct comparisons between our findings and those of previous meta-analyses were not straightforward.

The inclusion of only women with a history of hysterectomy is the most notable strength of our study, as it enhances the homogeneity of the target population. By focusing specifically on this subgroup, we were able to minimize potential confounding factors related to the presence or absence of a uterus. This targeted approach allows for a more precise analysis and interpretation of outcomes related to apical pelvic organ prolapse in women who have undergone hysterectomy. Consequently, our study provides valuable insights specific to this homogeneous population, which can contribute to a more accurate understanding of the effectiveness and safety of the studied interventions.

### 4.2. Future directions

As the field of surgical approaches for vaginal vault prolapse continues to evolve, a few topics for future research and improvement can be identified. This meta-analysis provides valuable insights into the comparative effectiveness and safety of different surgical techniques. However, there are still areas that warrant further investigation to advance clinical practice and patient outcomes.

Firstly, given the increasing prevalence of robotic surgery, future studies should focus on comparing robotic procedures with the currently established laparoscopic techniques performed for vaginal vault prolapse. Robotic surgery offers potential advantages, and evaluating its outcomes and benefits in comparison to traditional laparoscopic approaches will help elucidate its role and potential benefits. Anticipated progress is not limited solely to the realm of robotics, as the laparoscopic field also shows promise. Techniques like vaginal natural orifice transluminal endoscopic surgery (vNOTES) offer a hopeful outlook as a surgical solution for vaginal apical prolapse. By utilizing the vaginal pathway, this approach minimizes incisions and reduces the likelihood of scarring. This approach aligns with patient preferences for less noticeable surgical outcomes and quicker postoperative recovery. However, it is important to acknowledge that the successful adoption and progression of robotic surgery as well as vNOTES requires thorough training and expertise among surgeons. The goal should always be to tailor the surgical approach to the individual patient’s needs, rather than adhering strictly to a single method.

Secondly, long-term follow-up studies are needed to assess the durability of outcomes and the potential for recurrence or complications over time. While our meta-analysis included studies with various follow-up durations, extended, standardized, follow-up periods are crucial to determine the sustained effectiveness of different surgical interventions and provide more comprehensive information for both patients and healthcare providers.

Also, a standardized methodology to report outcomes and outcome measures is urgently needed to allow robust comparisons. Due to the high variety in outcome reporting by primary studies, our study only allowed comparisons of perioperative outcomes and of two, medium-and long-term outcome measures, namely UDI and IIQ. Although the primary papers included in this study evaluated various outcome measures such as The Australian Pelvic Floor Questionnaire (APFQ) (15), Defecation Distress Inventory (DDI) (15, 21), Pelvic Organ Prolapse/Urinary Incontinence Sexual Function Questionnaire (PISQ) (8, 15, 17), Female Sexual Function Index (FSFI) (18), Pelvic Floor Distress Inventory (PFDI) (17), Pelvic Organ Prolapse Distress Inventory (POPDI) (17), and Colorectal-Anal Distress Inventory (CRADI) (17), it was not feasible to conduct meta-analyses for those outcome measures because they were reported in isolated fashion, by only one or two studies. It is important to prioritize those outcomes in future studies to ensure that research is relevant to clinical practice.

Another interesting aspect arises from complications associated with mesh materials. In this study, we aimed to investigate the outcomes and complications associated with different suspension techniques in vaginal vault prolapse surgery. While we acknowledge the importance of examining graft-related complications and their comparative analysis between these techniques, it is crucial to note that our study’s focus was primarily on the specific outcomes and complications we could assess with the available data.

Graft-related complications represent an important area of concern in pelvic organ prolapse surgery, and their analysis could potentially provide valuable insights into the overall efficacy and safety of the suspension techniques. Unfortunately, due to limitations in data availability, we were unable to perform a comparative analysis of graft-related complications in this study.

We recognize this as a limitation of our work and believe that future research endeavours should prioritize the collection and analysis of data related to graft-related complications in the context of different suspension techniques.

Lastly, cost-effectiveness analyses are essential to evaluate the economic implications of various surgical approaches. Assessing the costs associated with different procedures, including initial costs, perioperative costs, and long-term follow-up costs, will aid in healthcare resource allocation and decision-making.

### 4.3. Limitations

Several limitations of this meta-analysis should be acknowledged. Firstly, the analysis was limited to the available studies identified through the multiple database searches and snowballing process, and potential publication bias cannot be completely ruled out. Secondly, the included studies exhibited heterogeneity in terms of study design, which may have influenced the overall results and comparability. Additionally, the quality and reporting of the included studies varied, which could impact the reliability and generalizability of the findings. Finally, the limited number of studies available for some specific outcomes may have affected the power and precision of the pooled analyses.

## 5. Conclusion

In summary, this meta-analysis highlights that LSCP does not present substantial advantages over VRP for apical prolapse after hysterectomy, while demonstrating certain advantages over ASCP in terms short term outcomes such as hospital stay, blood loss, pain, and ileus rates. These findings contribute to the understanding of the comparative effectiveness of different surgical techniques, assisting clinicians in making informed decisions regarding the most suitable approach for the surgical management of apical prolapse.

## Data availability statement

The original contributions presented in the study are included in the article/supplementary material, further inquiries can be directed to the corresponding author.

## Author contributions

RC: Conceptualization, Supervision, Writing – review & editing. M-PR: Conceptualization, Formal analysis, Methodology, Writing – original draf. AM: Writing – review & editing. CB: Data curation, Formal analysis, Writing – review & editing. CO: Data curation, Writing – review & editing. IN: Data curation, Writing – review & editing. DM: Supervision, Writing – review & editing.

## Funding

The author(s) declare that no financial support was received for the research, authorship, and/or publication of this article.

## Conflict of interest

The authors declare that the research was conducted in the absence of any commercial or financial relationships that could be construed as a potential conflict of interest.

## Publisher’s note

All claims expressed in this article are solely those of the authors and do not necessarily represent those of their affiliated organizations, or those of the publisher, the editors and the reviewers. Any product that may be evaluated in this article, or claim that may be made by its manufacturer, is not guaranteed or endorsed by the publisher.
